# Local initiative supports case isolation and contact tracing during a SARS-CoV-2 surge in summer 2020: a community case study in Antwerp, Belgium

**DOI:** 10.3389/fpubh.2023.1000617

**Published:** 2023-05-05

**Authors:** Kristien Verdonck, Stefan Morreel, Jef Vanhamel, Bea Vuylsteke, Christiana Nöstlinger, Marie Laga, Josefien van Olmen

**Affiliations:** ^1^Department of Public Health, Institute of Tropical Medicine, Antwerp, Belgium; ^2^Department of Family Medicine and Population Health, University of Antwerp, Antwerp, Belgium

**Keywords:** COVID-19, community case studies, case management, contact tracing, source identification

## Abstract

In Antwerp, Belgium's second largest city, a COVID-19 surge in July 2020 predominantly affected neighborhoods with high ethnic diversity. Local volunteers reacted and set up an initiative to support contact tracing and self-isolation. We describe the origin, implementation, and transfer of this local initiative, based on semi-structured interviews of five key informants and document review. The initiative started in July 2020, when family physicians signaled a surge of SARS-CoV-2 infections among people of Moroccan descent. Family physicians feared that the mainstream contact tracing organized by the Flemish government through centralized call centers would not be efficient in halting this outbreak. They anticipated language barriers, mistrust, inability to investigate case clusters, and practical problems with self-isolation. It took 11 days to start up the initiative, with logistical support from the province and city of Antwerp. Family physicians referred SARS-CoV-2-infected index cases with complex needs (including language and social situation) to the initiative. Volunteer COVID coaches contacted cases, got a contextualized understanding of their living conditions, assisted with backward and forward contact tracing, offered support during self-isolation, and checked if infected contacts also needed support. Interviewed coaches were positive about the quality of the interaction: they described extensive open conversations with cases. The coaches reported back to referring family physicians and coordinators of the local initiative, who took additional action if necessary. Although interactions with affected communities were perceived as good, respondents considered that the number of referrals by family physicians was too low to have a meaningful impact on the outbreak. In September 2020, the Flemish government assigned the tasks of local contact tracing and case support to the local health system level (primary care zones). While doing so, they adopted elements of this local initiative, such as COVID coaches, tracing system, and extended questionnaires to talk with cases and contacts. This community case study illustrates how urgency can motivate people to action yet support from people with access to resources and coordination capacity is vital for effective organization and transition to long-term sustainability. From their conception, health policies should consider adaptability of new interventions to local contexts.

## 1. Introduction

Worldwide, the COVID-19 pandemic has disrupted societies and healthcare systems since early 2020 (1, 2). In Belgium, the first wave of COVID-19 cases started in March 2020 and reached a peak in April 2020 (3). The Belgian authorities initially responded with a countrywide lockdown and suspension of routine non-acute care. Family physicians (FPs) were asked to set up infection clinics for medical triage to avoid overcrowding of emergency departments (4, 5). Despite these drastic interventions, the population was badly hit: during the first COVID-19 wave, Belgium reported national excess mortality rates that were among the highest in the world at that time (6). In May 2020, when the number of cases declined steeply, the authorities gradually released population-level control measures. These measures were replaced by broad SARS-CoV-2 testing policies, a centrally organized contact tracing (CT) system, and self-isolation requirements for positive cases. The aim was to switch from a population-wide quarantine to a quarantine for those who were infected and their contacts (7).

The World Health Organization (WHO) considers contract tracing an essential public health measure to control the spread of specific infectious diseases; it is the process of identifying, assessing, and managing people who have been exposed to an infected person (8). The rationale of contact tracing is to prevent onward transmission through the rapid identification and management of secondary cases that may arise after transmission from the index case. Contact tracing is closely linked to the isolation of the index case and to backward tracing, i.e., the search for the source of infection in the index case (9, 10). A recent systematic review into the effectiveness of contact tracing included six studies focusing on COVID-19, four of which found contact tracing interventions to be associated with improvements in at least one outcome of interest, i.e., case detection rates among contacts or at the community level, overall forward transmission, or overall disease incidence (11). Developing and implementing contact tracing strategies in the midst of a pandemic was a challenge for many countries. Belgium opted for a centralized approach, with call-centers, staff recruitment, and governance at the level of the regional governments. In those early stages, central capacity building was prioritized over engagement with local communities, an engagement that later turned out to be essential at all stages of the process (12).

Faced with a COVID-19 surge in specific communities in July 2020, a local bottom-up initiative was set up in the city of Antwerp with the aim to support case isolation and backward and forward contact tracing. In this paper, we describe the context in which this local initiative originated, document on-ground implementation experiences, and describe the perceived impact from the perspective of the early implementers. This local initiative can be considered as a case of a local response to a health crisis in an urban context. There is much to learn from adaptive responses in health crises as they provide a unique opportunity to reveal and study the structural gaps in the health system that such responses aim to fill. The Antwerp case is of particular interest because it illustrates an ongoing debate in health policy and systems research, notably that of the tension between a centrally coordinated “institutional” approach to respond to health emergencies, set up to be efficient (in our case, call-centers) vs. a decentralized and flexible approach that is often argued to be more effective and sustainable (in our case, community-embedded COVID coaches). By providing a detailed and rich account of how this local initiative emerged and evolved, our case provides insights into the conditions under which local and central approaches interact and may work more synergistically in the future.

To collect information, we purposefully selected and interviewed five key informants who were involved in the set-up and the implementation of the local initiative. We aimed to include participants with diverse backgrounds and roles in the initiative (FP, academic expert, project manager, COVID coach recruited and trained by the local initiative to perform contact tracing tasks). The interviews were organized online and took place between February and October 2021. The interviews unfolded in a conversational manner and lasted ~1 h each, guided by a semi-structured topic guide. The interviews were audio-recorded and transcribed verbatim in Dutch. We used an inductive coding approach to identify themes emerging from the data. To facilitate coding and analysis, we used NVivo version 10 (QSR International Pty Ltd., Cardigan UK). Where relevant and possible, we verified the information the interviewees gave us by checking other sources, i.e., documents and datasets published by Sciensano (National Institute of Public Health in Belgium), news media, and email conversations shared with us by key stakeholders. The study protocol was approved by the Institutional Review Board of the Institute of Tropical Medicine of Antwerp (reference number 1450/20). Before the start of the interviews, the participants provided oral informed consent which was audio-recorded.

## 2. Context

### 2.1. Community context

Antwerp is Belgium's second largest city, located in the region of Flanders. In 2021, 53% of residents had a migration background, making Antwerp a majority minority city. The city currently hosts a population of more than 500,000 inhabitants originating from 180 nationalities (13). The majority of Antwerp's residents of foreign origin stem from North Africa (14%, with Moroccans constituting the second-largest group after residents with Dutch nationality), West Asia (9%) and Eastern Europe (9%). People with a migration background are unevenly distributed across the city, with larger proportions in socio-economically deprived and densely populated neighborhoods, where informal working conditions and crowded housing are likely to facilitate SARS-CoV-2 transmission (14, 15). A qualitative rapid assessment conducted among ethnic minority communities in Antwerp identified the socio-economic and socio-cultural challenges in adhering to COVID-19 control measures, and showed that some migrant communities were inadequately reached by public health messaging (16).

### 2.2. COVID-19 surge in Antwerp in July 2020

**Figure 1:**
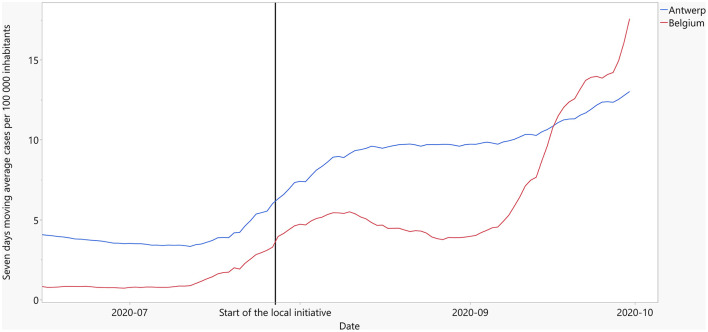
Trend in COVID-19 cases in Antwerp and Belgium (summer 2020).

The incidence of COVID-19 in the city of Antwerp followed the national trend of a steep first wave with a quick recovery leading to a very low number of cases in the beginning of the summer. However, in the middle of July 2020, FPs responsible for test centers in Antwerp noticed an increase in the number of cases (Figure 1). Based on initial explorative history-taking and informal forward and backward contact tracing, they suspected a large but localized outbreak in the Moroccan community, possibly linked to several wedding parties. These FPs feared that the centrally organized contact tracing would not be efficient in halting this local outbreak. They contacted the Flemish government, infectious diseases and epidemiology experts embedded in the Antwerp context, and the provincial authorities. Jointly they decided to complement the central approach with a local initiative. A few days later, provincial authorities took additional population-wide measures (night curfew and mandatory use of face masks) which were continued for 1 month. Halfway August 2020, the number of cases in the city of Antwerp decreased until September 2020, when they started to increase again as part of a nation-wide second wave (Figure 1). A chronological overview of relevant events is given in Table 1.

**Table 1:** Chronological overview of events relevant for the Antwerp local initiative, March 2020 to September 2020.

**Event**	**Level**	**Date**
There is evidence of local circulation of SARS-CoV-2 in Belgium.	Federal	3 March
A stringent lockdown is imposed.	Federal	14 March
Deaths due to COVID-19 reach a peak.	Federal	10 to 14 April
Governments decide to organize contact tracing at the regional level via professional call centers.	Federal	21 April
In other -even more local- initiatives in the city of Antwerp, FPs decide to organize contact tracing locally and train volunteers to support them.	Local (Antwerp)	End April
Large-scale testing is introduced: all people with symptoms of COVID-19 can now be tested.	Federal	1 May
The most stringent lockdown measures are gradually relaxed.	Federal	11 May
Centralized call centers start working.	Regional (Flanders)	11 May
FPs in Antwerp raise the alarm because they notice an increase in COVID-19 cases in specific communities.	Local (Antwerp)	10 July
The governor of Antwerp, city representatives, FPs, and experts hold a crisis meeting.	Provincial (Antwerp)	16 July
The local initiative starts to contact index cases referred to them by FPs.	Local (Antwerp)	27 July
Curfew and facemasks in public spaces are imposed in the Province of Antwerp.	Provincial (Antwerp)	29 July
A local initiative in another city in Flanders starts contact tracing and related support activities.	Local (Kortrijk)	1 August
The activities of the Antwerp local initiative are transferred to the corresponding primary care zone.	Local (Antwerp)	Early September

### 2.3. Health system and policy context

The Belgian health system covers 99% of the population with a broad package of services that are (partly) reimbursed. Health expenditure is around 10% of gross domestic product. The main source of health financing are social contributions. Health care services are generally delivered by private providers and hospitals, yet these are mainly affiliated with the national reimbursement system for their financing. People can get health care at the facilities and physicians of their choice (17). Belgium does not have a system of public health facilities where prevention and health promotion services are centralized.

Belgium has four different levels of governing power: federal (national), federated (regional), provincial, and local (municipalities) (18). Health-related responsibilities are divided among the federal level (financing, organization and professional regulation) and regional levels (disease prevention, health promotion, and the organization of primary health care, home care, and social care) (19). In the Flemish region, the Agency for Care and Health created 60 primary care zones in 2019, in order to plan and organize care on the local level for the population in their territory (20). Health care responsibilities of the provinces and municipalities are limited to matters of local interest and they act under the supervision of the regions. However, for crisis management, the federal government Ministry of the Interior takes the lead.

Infectious disease prevention and control falls under the responsibility of the regional health authorities. Yet, the delivery of preventive services is less clearly organized and fragmented across government-funded centers, primary care practitioners, hospital services, and a range of non-governmental and community organizations. In this system, contact-tracing, and case-isolation activities for notifiable infectious diseases such as tuberculosis have been traditionally taken-up by dedicated infectious disease control units of the regional health authorities, one per province. These units were (pre-COVID-19) relatively small and lacked capacity to respond to large-scale infectious disease outbreaks. The setting up of a new and centralized system for COVID-19 contact tracing needs to be understood against this organizational background.

**Figure 2:**
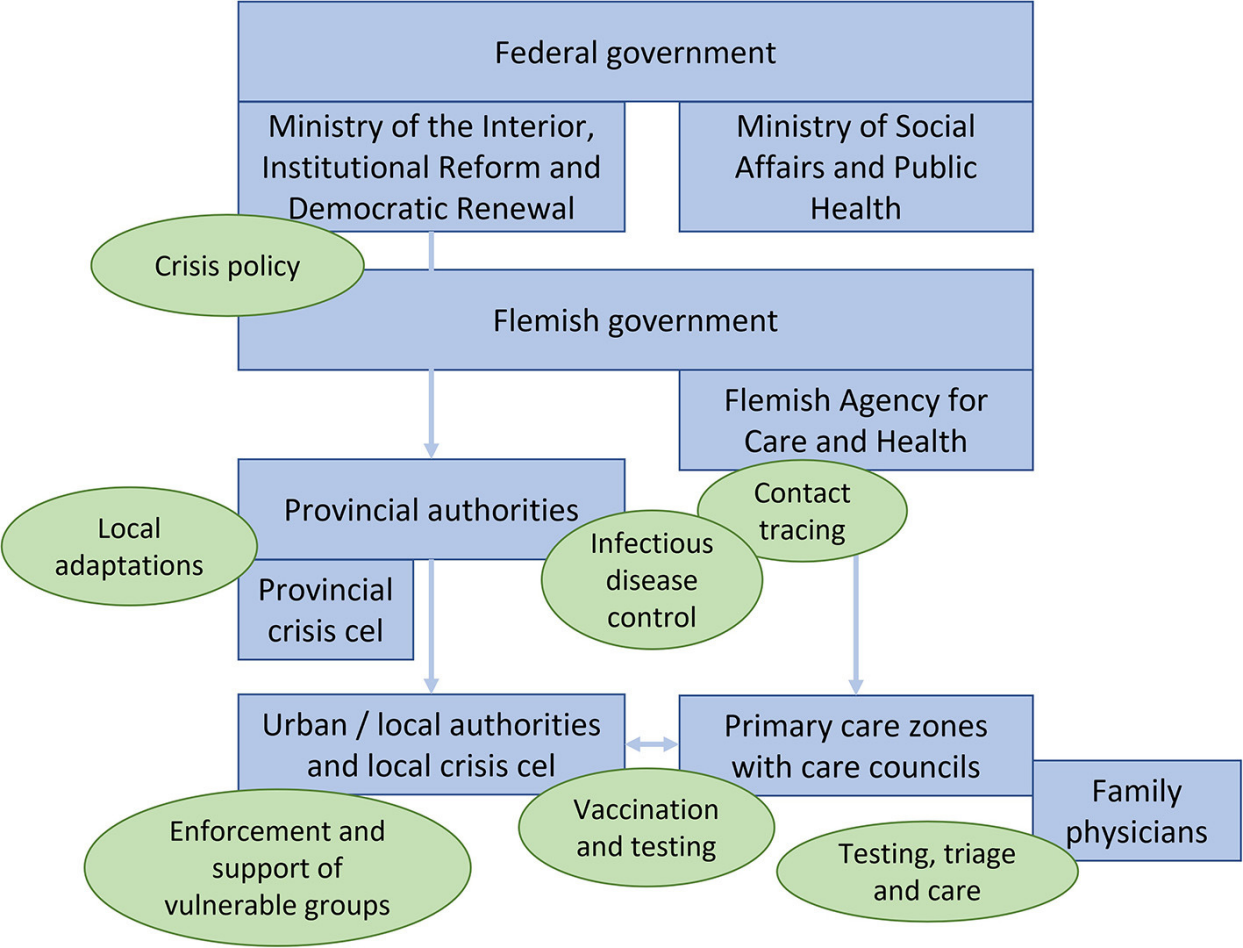
Different levels of organizations/authorities (blue fields) and their tasks (green fields) in the management of the COVID-19 crisis in Belgium in the summer of 2020.

From the start of the COVID pandemic, a crisis cell was formed at federal level, with a concertation committee including national and regional governments to determine the national COVID policies. Crisis cells were also set up at province and municipality levels with direct communications with the federal level. Figure 2 visualizes the organizations (rectangles) and tasks (ovals) at different levels. It turned out difficult to develop a coherent policy response across the different levels of government in a swift way: communication depended on regular inter-ministerial conferences and communication in different directions that was not institutionalized (21). This delayed response and created room for tension, further fed by different political parties ruling in government at different levels (19).

## 3. Details to understand key programmatic elements

### 3.1. The installation of the local initiative

FPs in Antwerp raised the alarm about a surge of infections in specific population groups and neighborhoods. The Flemish authorities were struggling to implement the central contact tracing system, which was heavily criticized in the media for its high cost, delays in contacting people, and suboptimal quality of telephonic counseling provided (22). The Flemish authorities failed to provide a quick and specific response to the rapidly changing and urgent on-ground reality experienced by FPs. The provincial authorities, by contrast, reacted immediately: the governor set up a crisis meeting with the local FP Board, academic experts, representatives of the city of Antwerp, and other crisis intervention stakeholders. The outcome of this meeting was the decision to set up a community-led local initiative with logistical support from the province and the city and financial support from the city alone. The local initiative entailed the recruitment and training of local volunteers as “COVID coaches,” who would take up backward and forward tracing and assess the need for support to people infected with SARS-CoV-2. Table 2 gives and overview of the key features of the local initiative compared to the centrally organized intervention.

**Table 2:** Key features of two different contact tracing strategies in Antwerp, Belgium in July 2020 to September 2020.

**Feature**	**Centralized contact tracing via call centers (22–24)**	**Antwerp local initiative^a^**
Period	Two years: May 2020 to May 2022	July 2020 to August 2020
Funder and cost	Regional government of Flanders. Estimated in 200 million euro for 2 years.	Urban and Provincial Authorities. Cost unknown, largely logistic support. The time investment of volunteers is not costed.
Staff profile	Professional call agents working from Brussels. These call agents do not know the people they call, and do not know the local context or background of people.	Volunteer COVID coaches living near the index cases. These COVID coaches have contact with the same FP, talk to several members of the same contact network, live in the same city, and may belong to the same community or speak the same language. Sometimes they know the people they contact (requiring professional confidentiality). Nursing students are remunerated as job students; other COVID coaches are not paid.
Focus of activities	Narrow: contact index cases, give information about COVID-19, call contacts. In2years, 2.3 million index cases are contacted. In low-transmission periods, agents have spare time, in high-transmission period, agents cannot follow.	Very broad: contact index cases, help them with listing contacts, answer their questions, try to identify sources of infection, support cases with self-isolation, provide flexible support depending on the needs of the cases and the expertise of the COVID coaches.
Target population	Call agents try to reach all individuals registered with SARS-CoV-2 infection as well the contacts they provide.	COVID coaches only contact index cases after referral by a FP. FPs refer relatively few cases, usually with complex needs (e.g., large households; people who do not understand Dutch, French or English; households including people with disabilities; undocumented immigrants).
Approach toward clusters of cases	People are approached as individuals. Hence, different call agents approach the different people of the same infection cluster (e.g., household members). This added to disconnect in the experience of people.	One index case is assigned to one COVID coach. This coach deals with the index case and all his/her contacts. This contributed to an integrated approach and a good overview of local connections.
Staff training	Call agents start working after limited training. Many lacked experience and expertise in conversations about health-related issues and contact tracing	Two hours training at the beginning. A training manual is developed in the first days of the initiative. But the selection through professional networks led to COVID coaches with relevant experience in health or social services (e.g., doctors, nurses, nursing students, teachers).
Direction of communication	One direction: call agents call index cases and contacts, who cannot call back.	More directions: COVID coaches work with specific cell phones for the project. This is needed for secure communication (via Siilo app). Apart from the index cases, they also call FPs, social services, and the coordinators of the local initiative among others. The people they contact can call them back.
Flexibility	Limited: call agents follow a script.	Pronounced: a coordinator of the initiative assigns cases to COVID coaches (e.g., taking language into account). COVID coaches adapt their approach to the cases.
Probability of identifying sources of infection	Low: initially, the call agents' script did not include questions about the likely source of infection. Some changes were made in September 2020.	Higher: COVID coaches try to identify the source of infection. They know the local context and the community. Also, the fact that one COVID coach contacts the different people involved in a transmission chain increases the probability of identifying common sources.
Contact with FPs	There is no interaction between call or field agents and FPs.	FPs refer cases to the local initiative. COVID coaches receive context information about the cases and can directly contact the FPs to request additional information or report back.
Calls and visits	If call agents cannot reach a person, field agents can do a home visit.	COVID coaches evaluate the situation and decide on a case-by-case basis what they can achieve by phone and when they will visit a person or a family.
Data collection and utilization	Data are collected systematically from laboratories into a central Belgian database, which then transfers data to the call centers. In the starting phase, there are many technical problems with transfer of data.	The focus is on support of people and on containment of the outbreak. Some data are collected to monitor the activities, not for further research. These data are organized per index case and contact network.

a Descriptions based on information provided by the interviewees involved in the Antwerp local initiative. If similar views are expressed in other sources as well, we have included references to these other sources in the table.

The following quote illustrates how previous bad experiences with the centralized call agent system motivated volunteers to participate in the local initiative as COVID coach:

“*I, who do know Dutch, found it difficult* [to deal with the agent from the call center]*, let alone someone who is older, or someone from a different culture and who does not know the language: that must be totally difficult. Then I thought: oh, I do want to join this initiative. Maybe we should visit some of the people.”* (COVID coach 1)

### 3.2. How the local initiative worked

The aim of the local initiative was to provide proactive and tailored support to those SARS-CoV-2-infected patients who needed it. FPs were informed about the initiative, and they were invited to refer SARS-CoV-2-infected index cases through a tele-based system (25), especially when they judged that the intervention of the centralized call center (described in Table 2) would not be sufficient. Interviewees reported how FPs mainly referred index cases with complex needs, such as people from Berber- or Arabic-speaking families with limited knowledge of Dutch, large households, families including people with disabilities, and foreign travelers who did not have a place to self-isolate or who had left the country while being SARS-CoV-2 positive. The following quote from a COVID coach illustrates how—through the referral system—the local initiative managed to reach certain types of vulnerable people, who otherwise fell through the cracks of the formal public health system:

“*The cases that were referred to me -I can only talk about my cases- were cases who were not reached by the contact tracers* [i.e., call agents]*, or there was no cooperation, or a language barrier, or the FP was worried. With me, there was no overlap* [with other contact tracing activities]. *I used to say to the person who referred the cases: “I mainly get the problem cases, the ones nobody else could manage.”* (COVID coach 1)

Based on the information provided by the referring FPs, a coordinator of the local initiative matched each index case to a COVID coach. These coaches were volunteers recruited from the professional and social networks of the initiators of the local initiative; and most of them were somehow connected with the affected neighborhoods or communities. They had a professional background in medicine, nursing, teaching, or other types of social work. They were in regular contact with the coordinators of the initiative and the FPs who referred cases; they were not supervised otherwise. Students who acted as COVID coach got remunerated for their work via a student contract with the city of Antwerp. For nursing students, the coaching work also served as a voluntary internship that could be considered part of their training. The other COVID coaches did not receive any financial or other incentives.

The task of the COVID coaches consisted in contacting the index case to support self-isolation and assist with backward and forward contact tracing. They worked in a relatively autonomous way: they talked to people on the phone and, if deemed necessary, they visited them. The same COVID coach managed the index case and the case's entire network of contacts. While referring FPs disclosed the diagnosis to the index cases, COVID coaches answered their questions and helped them list their contacts. Usually, the COVID coaches also helped the index case to call their contacts and kept in touch to check if any infected contacts also needed support. During the conversation with infected people, the coaches enquired about probable sources of infection. They tried to motivate the cases to self-isolate and provided practical assistance where possible. This assistance could take many forms, such as organizing food delivery, contacting a trade union for people in fear of losing their job, and getting a code required for SARS-CoV-2 testing of contacts. The coaches reported back to the referring FPs and the coordinators of the local initiative, who could take additional action if necessary (e.g., by reporting possible transmission events at a sheltered employment site or during wedding parties).

Through the ability to spend time with cases, COVID coaches were often able to identify complex social and practical care needs:

“*The psycho-social aspect was important. What is going on with these people? What do they need? Some people actually live in illegality. How do we deal with that? These are the people who are often referred to us. People without… yes… a national registry number and so on*.” (COVID coach 1)

“*For example, a single mom, and she cannot leave the house to get groceries: how do you deal with that? Then I had the community, yes, I contacted the community; look, there is a single mother with two children, including one with autism: who can do this or that? And people reacted: ‘yes, look, I can do the shopping, I'll drop it off at your place.' I actually went to drop it off at her door and then left.”* (COVID coach 1)

“*That was about a deaf-mute person of foreign origin who would, they said, have a brother or a cousin, they didn't know that very well, who could interpret. I agreed to meet them outdoors* [in a public place]. *I sat down with them very discreetly in an isolated corner and indeed, he was deaf-mute. He worked in a sheltered workshop where they always took off their face masks. (…) And at the same time, it turned out that the brother or the cousin, who was clearly quite sick, did not have a FP but needed to be tested. The problem was that back in July [2020], people without a FP could not get a code for COVID testing*. (COVID coach 2)

### 3.3. What was needed to start working

The local initiative was established on 16 July 2020. In the following days, an FP drafted the main principles and a volunteer with extensive management experience was appointed local initiative manager. The local council of the district of Borgerhout made an office available for face-to-face activities. The local initiative manager purchased some basic materials, such as disinfection alcohol, face masks, and any other personal protective equipment the COVID coaches might need. The coordinators and the coaches received a dedicated smart phone so that they could communicate with cases, contacts, and social and healthcare workers in a flexible and private way. All confidential communication took place through Siilo (Amsterdam, Netherlands), a free secure messaging app for healthcare professionals. Based upon individual needs, the COVID coaches were facilitated to perform their tasks: for instance, a bike to move around the city or a temporary place to stay so that they would not put their housemates at risk for infection. The city administration facilitated all contracts, premises, and materials, whereas the provincial authorities paid remaining bills. The first case was contacted on 27 July 2020, so it took 11 days to get started. Coaches received a basic 2-h online training, involving skills in motivational interviewing, a short technical training in the rationale and performance of contact tracing, and practical information about operational aspects of the program. A complete manual for COVID coaches was developed by the local initiative team and made available by the end of August.

### 3.4. Who were the volunteers

The local initiative team included one manager, two coordinators, three expert advisers (FP and epidemiologists), and ~20 COVID coaches. Together, they constituted a multidisciplinary team with diverse experience and complementary skills in first-line health care, management, engagement with minority populations, and epidemiology and control of infectious diseases in Belgium or in low-income settings. They got involved because someone in their network invited them to participate. Most were on sabbatical, parental leave, summer holidays, or recently retired, which allowed them to invest time. Their motivation to join was rooted in personal or professional experience. Some volunteers with a Moroccan background, for instance, felt that their community was not treated fairly by the news media and the call centers. Some infectious disease experts joined because they were convinced that a local and flexible approach would be more effective than the centrally organized call centers. Regarding the COVID coaches, the bulk of the work was done by approximately five people from the pool, who were most available and had the language skills needed.

### 3.5. How the coaches experienced their work

The coaches we interviewed experienced sometimes difficulties establishing the first contact, for example when index cases spent a lot of time away from home or when their contact details were inaccurate. However, they were in general very satisfied with the process and outcome of the interaction. They described long and open conversations and could not remember people who refused to collaborate. But despite this willingness to collaborate and the support provided by the local initiative, several interviewees mentioned that in some of the affected households, self-isolation was very difficult or even impossible. The following quotes illustrate the COVID coaches' way of working, their person-centered approach, their trust bond with the patient, and their perceived barriers.

“*Well, if I had a phone number, I first called them. But sometimes we didn't get a phone number. Then I went there and rang the doorbell, and nine out of ten times, people just let me in.”* (COVID coach 1)

“*In the past, I have worked for a while with Child and Family services, so I know the network of social services in Antwerp, where people can go for help or food parcels, to get things delivered at home, or to get help at home (…). If I then noticed a child that did not look healthy and if people said they did not have the money to go to a doctor… well there are places … Médecins du Monde, or local FP practices where I would refer them to. I then also made the appointments for them* [Interviewer:] *And did you have to do that often?* [Interviewee:] *Yes, unfortunately. And I usually made my planning thinking: look, I can do that in half an hour or an hour, but usually it's 2 or 3 h.”* (COVID coach 1)

“*I once tried to contact a case together with an Arabic speaker. Together on the phone…to try to find someone. Sometimes it is a matter of: how can we find that person because the phone numbers are wrong? I once had, that was later in the year, a prostitute who was impossible to find. Eventually, I took my bike and drove by that place and thought, do these people actually live here? Well, sometimes people just give fake phone numbers and addresses. Some of them were just impossible to find.”* (COVID coach 2)

“*Such a call could sometimes take 2 h, because people would suddenly start to tell me about their lives and all their problems. That put me in a position to report situations to the coordinator and his team: look, in this social housing context something is going wrong. Or for example interim workers who had to quarantine and consequently lost their benefits because they did not manage to contact their trade union. And such situations came on top of all the rest*. [It explains] *why people had a hard time going into quarantine. But in such long conversations, you could gain an enormous amount of trust and you also got an enormous amount of information, in terms of risk contacts or situations that were indeed unsafe*.” (COVID coach 2)

“*I was impressed that one of the volunteers, Moroccan, had managed to get an illegal immigrant to call back. I found that… So, someone has COVID, that person does undeclared work in (…), and he says, yes, I work there, together with another undeclared worker, an illegal immigrant. And he managed to convince him (the illegal immigrant) that the contact tracing was not dangerous, that it would be more dangerous if he became ill. So, he came in and got tested.”* (manager/coordinator)

The following patterns emerged from the interviews with two COVID coaches and were confirmed by three respondents who had taken up other roles in the local initiative. First, coaches were creative and perseverant in establishing contact with the cases. Second, they used diverse professional and social skills to engage with the cases; hence, the type of support the cases received varied considerably depending on the coaches' expertise. Third, the coaches were generally satisfied with the way in which they had been able to fulfill their role and with the quality of the interaction with cases, FPs, and with the coordinators of the initiative.

### 3.6. What was the perceived impact

#### 3.6.1. Output

The local initiative wanted to collect some basic data on each index case but in the end, no structured records were created. COVID coaches only informally reported their findings to the FP and the local initiative manager. Between 27 July and 24 August 2020 (5 weeks), the local initiative handled 53 index cases of whom 47 (89%) could be reached. These 47 index cases reported 108 household and 87 other contacts (mean of 4 contacts per index case). In 19 cases (40%), a probable source of infection could be identified: a family gathering or party (*n* = 7), at work (*n* = 4), or in diverse other settings (*n* = 8). This contrasts with the central contact tracing at that time: ~60% of index cases were reached, half of whom give contacts. This increased later on (>90% in September 2020) (26). In September 2020, when the Flemish government assigned the tasks of local contact tracing and case support to the local health system level (primary care zones), the local initiative was disbanded. The COVID coaches remained active until March 2022 when the crisis phase of the COVID-19 pandemic came to an end in Belgium and almost all specific measures were discontinued.

COVID coaches reported many barriers and gaps in unmet support needs. This included practical difficulties in adhering with isolation or quarantine measures due to housing (e.g., crowding), working (e.g., fear of losing an undeclared job), or family conditions (e.g., caring for family members with disabilities). Another issue that emerged was mistrust and tensions related to the way in which specific communities were portrayed in the news media. Finally, the respondents mentioned hesitancy to name contacts in certain communities because of concerns about the possible consequences (e.g., interference with travel plans and accusations of causing transmission).

#### 3.6.2. Impact on COVID-19 epidemic and on the health system

Although some of the coaches believed that the local initiative helped to reverse the COVID surge in Antwerp in August 2020, the experts we interviewed considered that the low number of index cases referred to the local initiative prevented it from having a meaningful impact on the course of the outbreak. Several interviewees struggled to understand the low referral rate, and thought of the following reasons: (1) FPs may see their role as being a doctor for individual patients and are not used to support households or communities; (2) some FPs may not have been aware of the initiative or they may have been deterred by the administrative and other burden of referring patients; and (3) there may have been a lack of clear division in roles and responsibilities between COVID coaches and FPs (FPSs may have just continued handling these cases themselves as they were the familiar point of contact for patients anyway). This is supported by Figure 1 in which no clear decline in the number of cases occurs after initiation of the local initiative. Nevertheless, the local initiative is perceived to have had a significant impact on the health system response in a broader sense. From the interviews, we identified several elements in the implementation that contributed to its functioning and thus to outcomes; these are listed in Table 3. In addition, the respondents mentioned the transfer of experience to the primary care zones, via people (some COVID coaches and experts continued to be involved) as well as training materials. The network that was established in this early phase was institutionalized in an Antwerp COVID advisory board with strong collaboration between primary care zones, the city of Antwerp, and various other actors. Moreover, together with similar initiatives in two other Flemish cities, this local initiative publicly emphasized the importance and feasibility of local community involvement in COVID containment. As these initiatives got broad media coverage, they probably influenced policy, contributing to the fact that local primary care zones now take up a role in contact tracing and support of self-isolation in the entire Flemish region.

**Table 3:** Overview of mechanisms mentioned as facilitators or barriers to success by people involved in the local initiative, with illustrative quotes.

**Mechanism**	**Quotes**
**Mechanisms mentioned as facilitators of success**	
Initiative set up by a combination of people with drive, expertise, network, time	“*I watched the sessions of the corona commission in parliament that were broadcasted on TV, where (person A—an epidemiologist) highlighted the importance of proper contact tracing and proposed to involve volunteers. So, I sent an e-mail to (A) saying, look, I'm available, not for medical work, but for things that have to do with coordination. And (A) passed my name on to (person B—a FP), and (B) was the one who had sounded the alarm to the governor of Antwerp, that something needed to be done. (B) called me and really wanted me to take up the coordination, because I think (B) was disappointed with the attitude of government officials.”* (manager/coordinator)
	“*And (B) said: we need people who know the community, who can go there. Ok. So, I phoned the general director of the (…) college in Antwerp, from the car, still that Saturday. (…) And she said, ah that's a very good initiative, of course I'll take care of it. She immediately phoned the head of the nursing school. On Sunday, I got the head of the nursing school on the phone, and I explained what we were looking for. And on Monday already, (…) I got a phone call from a Moroccan nursing lecturer. I explained to her what profile I was looking for and she found a few additional volunteers.”* (manager/coordinator)
Collaboration with urban authorities: very local level, concrete, practical, swift	“*I called or mailed, and I said: I need thís. And then we could just go there and pick it up. And the bills were settled by the City of Antwerp, under their emergency plan, I suppose. That was good; otherwise, it wouldn't have worked. I couldn't have engaged those students, who always had to be on call (…). If we hadn't been able to give them a contract, it would have been completely voluntary work. But these students had to work to pay their studies; they were not the ones who were doing nothing. So, that is how things moved. If I said at 10 o'clock in the morning: I need five cell phones, then I had five cell phones at 3 o'clock in the afternoon. The advantage was that Antwerp is a very big city, so they have a big administration. I don't know if every small town has surplus cell phones. And the civil service of Antwerp was really good; that was really striking*.” (manager/coordinator)
Case managers from the community (language, participation)	“*I think we were the only ones who had field agents at that time. In Flanders, they were in the process of recruiting them. And above all, we had really recruited them in function of the group in which the problem was situated, and I think that was the difference. They could all speak Berber and Arabic. They were very very motivated because it was about their own family and their own friends.“* (manager/coordinator)
Insights by FPs and case managers: quick and to the point—feedback loops short	“*I always informed the FP; I always made a small report. And then if the FP asked me if it would be possible to drive by and check on the family, I would take my car and go there.”* (COVID coach 1)
**Mechanisms mentioned as barriers to success**	
Combination of fast action and confidentiality vs. data collection (reporting and monitoring)	“*If you go in with a notebook and all sorts of things, it comes across as if you've come to check up on things. And that's what I definitely wanted to avoid. I always had a small piece of paper with me, just a piece of white paper and a pen, and I would say: I'll just take a few notes. I would not be sitting there with a notebook or a laptop. I was asked to do that, but I said no.”* (COVID coach 1)
	“*And then we saw that the FPs were actually referring very few patients. But we were sure there were many more vulnerable families out there, so the system of referral by FPs did not work very well. (…) For me as an FP, it's a bit difficult to understand that you get such an opportunity and then you don't make much use of it. I think… yes… FPs may not see it as their task. They see their role as being a doctor for the individual patient. So, the patient calls you and you help them, but then, making the link with the whole family, or having to help the neighborhood: FPs don't really do that.”* (expert adviser)
Limited skills and availability of volunteers to be fully deployed	“*For the other volunteers, it was a bit complex, we always had to look: when are they available. There were people who had a week's holiday (…) but after that week they dropped out. Then there was someone from* [another town nearby]*, and she was asked to help there, because in the meantime other activities were starting.”* (manager/coordinator)
Limited collaboration with Flemish Agency due to availability, perceived needs and institutional arrangements not allowing flexibility	“*So, these FPs did not get any reaction from the Agency for Care and Health in Brussels. And there they were stuck with a lot of positive patients who they knew would either not answer their phone -at that time calls still came in from a private number or a strange number; how many people answer the phone- or they would not understand the call agent. They speak Arabic or I don't know what language, that would not work*.” (expert adviser)
	“*And some people of the higher-level bureaucracy—I could not help noticing—really were on holiday. I wanted to send an email to ask: if we identify a cluster of cases, where we should report that? Ah yes, here is an email address. You send a message to that email address, and you get an automatic out of office reply: “I am on holiday and your emails are not forwarded.” Health authorities in the throes of a pandemic… I cannot understand that.”* (manager/coordinator)

## 4. Discussion

The community case study in this paper describes how a local contact tracing and isolation initiative emerged, was implemented, and transferred in the COVID-19 pandemic context. The aim was to complement the central contact tracing system with locally embedded contact tracing and support to infected people by locally embedded volunteers. The results were: improved success rate of contacting infected people and their contacts, customized social and practical support to people in need and creating trust between infected people and providers. The successful implementation was facilitated by a sense of urgency of a team with sufficient agency and local embeddedness to pool and organize resources in a short timeframe. The low referral of cases to the team led to the quantitative output being modest, with an average of 2.6 cases contacted per COVID coach over 4 weeks.

The rapid and efficient organization contributed to the perception of efficacy. The implementation started from a strong sense of urgency from people with sufficient agency to act. Most community initiatives in outbreak settings emerge with local leaders, organizations and key individuals who observe that the formal response is either not present or not sufficiently adapted to the local context (27). Also in our case, a similar gap was felt early on by active and responsive people working on the ground and local and provincial leaders who quickly found each other in a common goal and plan, increasing collective responsibility. The rapid development of guidelines, a flat organizational structure with a local contact tracing system parallel to the central system, and the mobilization of a pool of volunteers and logistic support facilitated rapid implementation, contributing to the perception of a rigorous and efficient system. Our findings concur with the analysis of success factors for local COVID initiatives in other settings: shared responsibility, co-production of systems, and perceptions of good management (28).

The COVID coaches experienced that their embeddedness in the community allowed for a rapid response and that home visits allowed to address social, economic, and cultural barriers, and facilitated the creation of trust necessary for motivation of people. Initiatives in other places also show that minimizing delays in the first contact and addressing nutritional, financial, and housing needs increases the likelihood of successful quarantine and isolation. Moreover, the combination of tasks—information, contact tracing, support, and referral to care—by the same person increased trust and engagement of people with the coaches (29). Speed and an integrated approach are crucial.

After local implementation, the transfer to an enduring model entails new dynamics. While a crisis motivates key persons and volunteers to act as driving forces, sustained engagement of people needs other incentives (29). The support from people with access to resources and coordination capacity facilitates coordination and integration into health system structures and processes (30). The Antwerp initiative was integrated into the mainstream epidemic policies. The Flemish government delegated the tasks of local contact tracing and case support to local health system level (primary care zones) and provided resources for hiring staff. Many elements of the local initiative's approach were adopted, such as the COVID coaches, the tracing system, and the extended questionnaires to talk with high-risk contacts and patients. The local initiative therefore had an impact beyond its original scope. More specifically, the new pandemic policy of Belgium (October 2021) identifies the mandates and responsibilities in a pandemic context. In this policy, the mandate of local authorities to develop contextual responses, such as in the local initiative, has become stronger and more explicit.

COVID coaches can be regarded as a kind of community health workers. Programs involving community health workers are relatively well-documented in low- and middle-income countries, in settings where there is a shortage of professional health workers, especially in the domains of mother and child health and control of infectious diseases (31–33). Recently, the concept of outreach through community health workers has also gained attention in Belgium and other high-income countries, with the aim to reduce inequities in access to and outcomes of care (34–36). In Belgium, there is an ongoing national pilot initiative involving community health workers with the goal of improving access to primary care for people living in socially vulnerable conditions in an urban context (37).

The review of this and other case studies provides lessons and practical implications for future bottom-up initiatives to address an outbreak in an urban context. Such case studies can contribute to practice-based evidence (as opposed to evidence-based practice): they convey key details “from the field” that may be useful for practitioners in similar situations. The first lesson is that bottom-up initiatives tend to complement gaps in a formal “one-size-fits-all” system. For instance, national response strategies often do not address the variation and diversities in urban communities (38). Volunteers from the local communities who know the neighborhood and who are trained to do multiple tasks are more effective in reaching people and in building trust so to do their work effectively and satisfactorily. This case in an urban setting, where capacity and agency are concentrated demonstrates that a local initiative can be developed in a rapid way. Although we did not study this, a hypothesis to be examined is whether the dynamic social environment of cities enables such local initiatives faster than in other areas.

Another key lesson is that while urgency can leverage a critical amount of energy among people to undertake action, the succeeding process of implementation plays a part in the feeling of shared responsibility and of success among those involved that is important to sustain. The energy, flexibility and local connect characterize local initiatives in their initial stage. If local authorities in future contexts aim to nourish such initiatives, they need to prepare a strong organization team to support volunteers and sustain engagement beyond the crisis moment. Local authorities also need to connect with health system decision-makers at higher levels to facilitate the transition to a long-term sustainability. The COVID-pandemic has taught us that multi-level governance with two-way feedback along the chain of community-based initiatives, local authorities, and central-level decision-makers is important to organize efficient and adaptive responses to crises.

## 5. Acknowledgment of any conceptual or methodological constraints

The current paper has several limitations. First, the target population of the local initiative (families with SARS-CoV-2 cases) was not interviewed. During the implementation, confidentiality proved to be very important. Afterwards, when the local initiative was transferred to the mainstream epidemic policies and we wrote the study protocol, we could not obtain consent anymore from the original target population. Second, although we aimed for diversity in the interviewees, the number of interviews (five) was rather low. With regard to the gap in public health services that led to the initiative, our different sources (respondents, news media, email communication) largely coincided and we moved toward saturation. The descriptions of the start and the operationalization of the initiative were also consistent. We did not reach saturation, however, for themes related to the sustainability and impact of the initiative because respondents did not agree or did not know. Third, the interviews were performed a few months after the initiative and this article was written more than 1 year later which might have led to a recall bias. Finally, author SM was one of the FP's that raised the alarm and was involved as one of the expert advisers in the initiative. For these reasons, the interviews were executed by author KV.

## Data availability statement

The datasets presented in this article are not readily available because giving ethical and privacy considerations, the studied data is not available for other researchers. Requests to access the datasets should be directed to tverdonck@itg.be.

## Ethics statement

The studies involving human participants were reviewed and approved by Institutional Review Board of the Institute of Tropical Medicine of Antwerp. The patients/participants provided their written informed consent to participate in this study.

## Author contributions

KV performed the interviews, inputted them in the software, curated, and analyzed them. Together with SM and JO, KV wrote a first draft which was reviewed and edited by all authors. Visualizations were made by authors SM and JO. SM and KV were responsible for the project administration. All authors were involved in the conceptualization, supervision, and the methodology of this paper. All authors contributed to the article and approved the submitted version.
